# Advances in Understanding *Leishmania* Pathobiology: What Does RNA-Seq Tell Us?

**DOI:** 10.3389/fcell.2021.702240

**Published:** 2021-09-01

**Authors:** Tamara Salloum, Sima Tokajian, Robert P. Hirt

**Affiliations:** ^1^Department of Natural Sciences, School of Arts and Sciences, Lebanese American University, Byblos, Lebanon; ^2^Faculty of Medical Sciences, Biosciences Institute, Newcastle University, Newcastle upon Tyne, United Kingdom

**Keywords:** *Leishmania*, RNA-Seq, gene expression, promastigotes, amastigotes, macrophages, skin lesions, metatranscriptomics

## Abstract

Leishmaniasis is a vector-borne disease caused by a protozoa parasite from over 20 *Leishmania* species. The clinical manifestations and the outcome of the disease vary greatly. Global RNA sequencing (RNA-Seq) analyses emerged as a powerful technique to profile the changes in the transcriptome that occur in the *Leishmania* parasites and their infected host cells as the parasites progresses through their life cycle. Following the bite of a sandfly vector, *Leishmania* are transmitted to a mammalian host where neutrophils and macrophages are key cells mediating the interactions with the parasites and result in either the elimination the infection or contributing to its proliferation. This review focuses on RNA-Seq based transcriptomics analyses and summarizes the main findings derived from this technology. In doing so, we will highlight caveats in our understanding of the parasite’s pathobiology and suggest novel directions for research, including integrating more recent data highlighting the role of the bacterial members of the sandfly gut microbiota and the mammalian host skin microbiota in their potential role in influencing the quantitative and qualitative aspects of leishmaniasis pathology.

## Introduction

*Leishmaniasis* is caused by a parasitic protozoan carried by over 90 sandfly species which are known to transmit more than 20 species of *Leishmania* parasites to humans, through either zoonotic or anthroponotic infection cycles (Ready, 2013; Rostamian and Niknam, 2019; World Health Organization [WHO], 2019). Three main forms of the disease exist and range in severity from mutilating cutaneous leishmaniases (CL) causing skin lesions and ulcers, mucocutaneous leishmaniasis (MCL) leading to a partial or total destruction of mucous membranes of the nose, mouth and throat and visceral leishmaniasis (VL), also known as kala-azar, affecting the spleen and liver and causing mortality in over 95% of untreated cases (World Health Organization [WHO], 2019).

**FIGURE 1:**
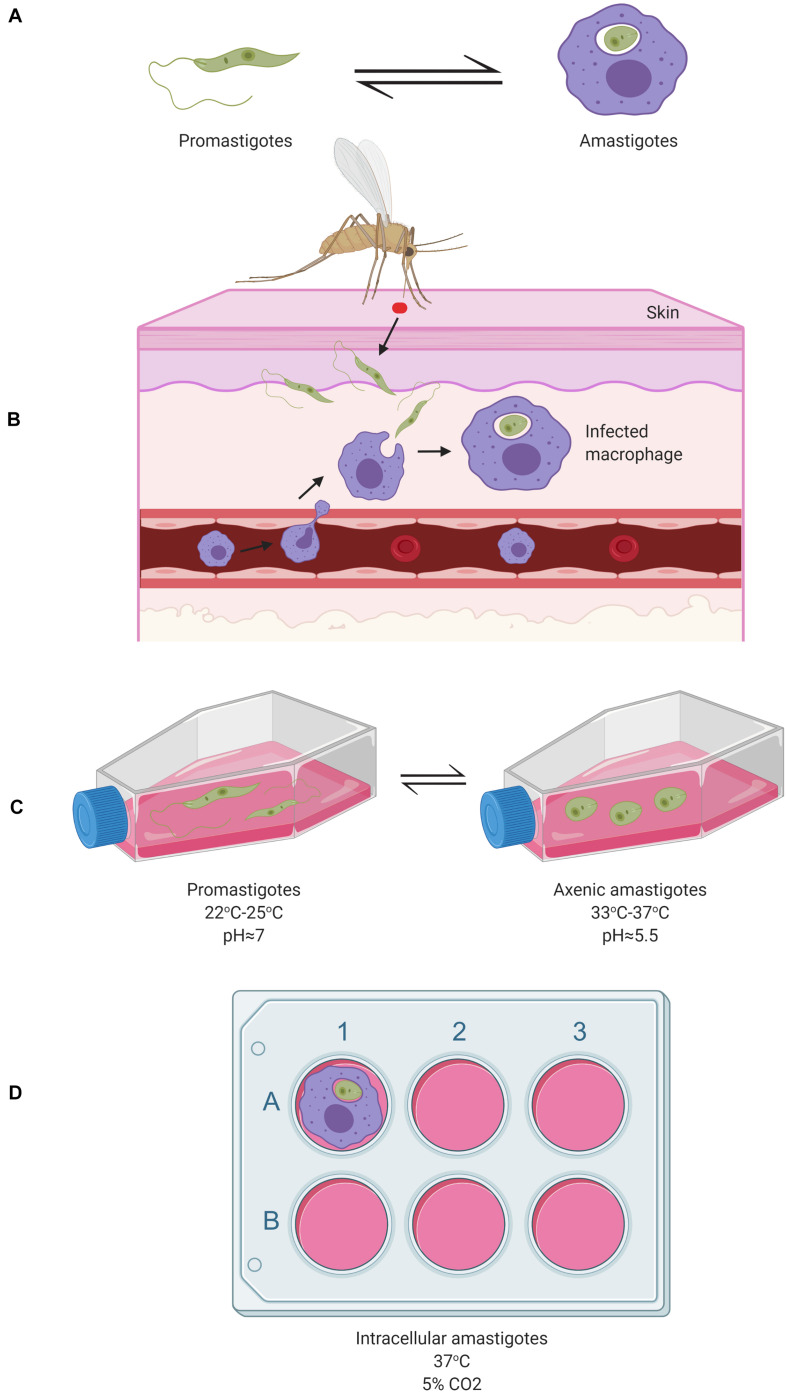
Schematic representation of the two major *Leishmania* cellular forms during their digenic life cycle *in vivo* and their study in corresponding *in vitro* model systems. **(A)** The flagellated motile promastigotes form (left) in the gut of a sandfly and the round intracellular amastigotes form residing inside infected mammalian macrophages (right), represent the two major cellular forms of *Leishmania* species. **(B)** Under *in vivo* conditions, sandfly vectors inject *Leishmania* promastigotes into the host’s skin following a blood meal which are then taken up by macrophages where they differentiate into amastigotes; **(C)** Under *in vitro* conditions, axenic promastigotes can be maintained in cell culture flasks in a rich, 10%- Fetal Bovine Serum (FBS)-supplemented medium (e.g., RPMI 1640, Schneider, Grace, or M199) at 22–25°C (left). These can be differentiated *in vitro* into axenic amastigotes in a medium at an acidic pH∼5.5 (Achieved through the addition of HCl) at 33–37°C (right) (Teixeira et al., 2002); **(D)** Macrophages (e.g., from human or mouse), can be seeded in 6-well cell plates in RPMI media supplemented with 10% FBS at 37°C to which the parasites are added resulting in macrophages infection within 4 to 24 h. More detailed aspects of the *Leishmania* life cycle are reviewed by Sunter and Gull (2017). Image created with BioRender.com.

*Leishmania* parasites, when alternating between the sandfly vector and the mammalian host, have two major corresponding life stages: promastigotes living in the sandfly’s gut and amastigotes residing inside mammalian macrophages (Figure 1). Following a blood meal, the sandfly ingests a macrophage containing *Leishmania* amastigotes. Once liberated from the macrophage inside the sandfly midgut, amastigotes differentiate into procyclic promastigotes. Then, the procyclic promastigotes become nectomonad promastigotes, which are able to cross the protective peritrophic matrix of the sandfly gut and attach to the microvilli of the epithelial cells of the midgut (Bates, 1994; Gossage et al., 2003; Bates, 2007; Sunter and Gull, 2017). From there, they can migrate to the thoracic midgut and stomodeal valve where they differentiate into leptomonad promastigotes. The leptomonad promastigotes can eventually differentiate into either haptomonad promastigotes, which attach to the stomodeal valve, or metacyclic promastigotes, which are the mammalian infective form. Metacyclic promastigotes are transmitted to the host by the sandfly during the next blood meal (Bates, 1994; Sacks et al., 2000; Rogers et al., 2002; Bates, 2007; Dillon et al., 2015a).

**FIGURE 2:**
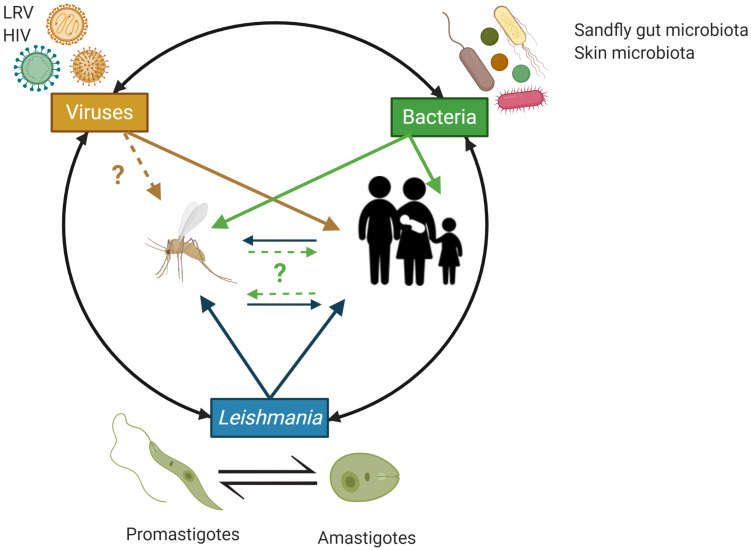
Schematic overview of the potential trans-kingdom cross talk between *Leishmania*, viruses (including *Leishmania* RNA virus – LRV) and bacteria from the implicated animal hosts microbiota and their impact on the human host. Animal hosts (here, sand flies and humans) are increasingly recognized to be supra-organisms made of animal cells/tissues/organs and their associated microbiota (e.g., associated with the human skin and mucosal surfaces) that through complex, continuous and highly dynamic interactions contribute to the host phenotype in both health and disease. In the context of infections by *Leishmania*, initiated by the bite of a sand fly, bacteria from the insect gut microbiota, the human skin microbiota and *Leishmania* RNA virus (LRV), which can infect *Leishmania*, have all be implicated in regulating the inflammatory tone associated with the infection by *Leishmania* and by doing so to the various pathobiologies associated with *Leishmania* infections. HIV is also indicated as AIDS can facilitate/worsen the pathobiology or *Leishmania* infections through weakening the host immune response and represent an important case of co-infections with negative impact on the human host. Image created with BioRender.com.

The diverse pathologies associated with *Leishmania* infections develop following excessive inflammatory responses by the infected tissues due to dis-regulated immune responses, which are increasingly appreciated to be triggered by complex network of interactions between the parasites, host immunocytes, the bacteria from the human skin microbiota and sandfly vector gut microbiota in addition to *Leishmania* RNA viruses (LRV) known to infect some *Leishmania* species (Ives et al., 2011; Brodskyn and de Oliveira, 2012; Gimblet et al., 2017; Cruz and Freitas-Castro, 2019) (Figure 2). Understanding the complex interplay between these factors involved in the host-parasite-microbiota interactions during *Leishmania* infection is crucial to refine the design of *in vivo* and *in vitro Leishmania* infection models and assays to study the biology of these parasites in more details and to eventually develop more effective prophylactic and therapeutic strategies with minimal or no side effects (Figure 2).

Although numerous studies have employed microarrays and RT-PCR to profile gene expression changes in *Leishmania* and host macrophages following infection (McNicoll et al., 2006; Srividya et al., 2007; Guerfali et al., 2008; Lin et al., 2008; Kumari et al., 2008; y Fortéa et al., 2009; Alcolea et al., 2010, 2011; Adaui et al., 2011; Probst et al., 2012; Rabhi et al., 2012, 2013; Beattie et al., 2013), global RNA sequencing (RNA-Seq) analyses emerged as a powerful methodology to generate a more global and systematic analyses of the transcriptome of the parasite and relevant cells from the concomitant insect vector and mammalian host. This review focuses on RNA-Seq based analyses of the transcriptome of *Leishmania*-hosts interactions and summarizes the main findings extracted using this technology. This includes the more detailed understanding of the interactions between human macrophages and *Leishmania* parasites and the differentiation of the parasite along its various cellular forms characteristic of its complex life cycle. By doing so we will highlight current caveats in our understanding of the parasite’s pathobiology and speculate on how RNA-Seq investigations will be able to further contribute at reducing our knowledge gap on the molecular and cellular basis of host-parasite interactions among these fascinating and complex parasites along with their microbial endosymbionts (e.g., LRV) and microbial neighbors (insect gut and mammalian skin microbiotas). This will integrate the more recent insights into the potential role played by other microbes associated with *Leishmania* environment, including bacteria members of the insect gut microbiota and the mammalian host skin microbiota.

## Control of Gene Expression in *Leishmania*

*Leishmania* and related trypanosomatids have unusual mechanisms to control gene expression (Clayton, 2016). Little transcriptional regulation appears to exist as non-functionally related genes arranged in large clusters are constantly transcribed into long polycistronic precursor RNAs (Martıìnez-Calvillo et al., 2003). Large polycistronic transcription units (PTUs) may be comprised of more than hundred genes with no obvious/apparent functional relationship (Beverley, 2003; Ivens et al., 2005). Kinetoplastid parasites rely almost exclusively on post-transcriptional gene regulation because of their constitutive transcription of RNA polymerase II (pol II)-driven polycistronic gene arrays (de Pablos et al., 2016). Accordingly, RNA binding proteins (RBPs) act as primary gene regulators and are overrepresented in the proteome (de Pablos et al., 2019). RNA binding proteins dynamically bind to mRNA forming ribonucleoprotein complexes (mRNPs) and regulate the trafficking and processing of mRNA molecules from synthesis to decay (Gehring et al., 2017). Moreover, it has been shown that in *Leishmania mexicana* parasites, mRNA levels are not a strong predictor of whole cell RNA expression or RNA binding potential of encoded proteins (de Pablos et al., 2019). Thus, the mechanisms mRNA stability, decay or translation are controlled in near absence of transcriptional regulation are still not fully elucidated (de Pablos et al., 2016).

Following transcription, precursor transcripts are processed into mature mRNAs coding for individual proteins through an unusual process known as *trans*-splicing (Matthews et al., 1994; Liang et al., 2003; Kramer and Carrington, 2011). During *trans*-splicing at specific positions, a polycistronic precursor is removed and a 39 bp long mini-exon is added to the 5’end of all mRNAs. The added mini-exon is also known as the spliced leader (SL) (LeBowitz et al., 1993). Then, a poly-A tail is added to the 3′-end of the mRNA. As such, the polyadenylation of the upstream gene is directed by and coupled to the *trans*-splicing of the downstream gene (LeBowitz et al., 1993). The mRNA is then exported to the cytoplasm where it can be recognized by the translation machinery by a highly modified 5′-cap structure on the mini-exon part (Freire et al., 2017). Notably, despite of the polycistronic transcription approach being used, adjacent genes often do not have the same levels of gene expression (Rastrojo et al., 2013). Indeed, some collinear mRNAs can have highly distinct steady-state expression levels (Rastrojo et al., 2013).

Despite these challenging and interesting/unusual features of the *Leishmania* transcriptome, variation in mRNA abundance has been observed between different life stages of the digenic infection cycles where mRNA levels were not considered as a strong predictor of the whole cell expression and protein levels (Lahav et al., 2011; de Pablos et al., 2016, 2019).

## Advantages and Limitations of RNA-Seq Technology

Global RNA sequencing and transcriptomics analysis illustrates significant variations in gene expression occurring during host infection with *Leishmania*, its survival inside the sandfly vector gut and during its differentiation between its two life forms as amastigotes and promastigotes. The implementation of RNA-Seq for studying gene expression in *Leishmania* culminated in a rapid expansion of the available knowledge with regard to the host parasite interactions, the existing variations, the relationship between the parasite and its vector and the differentiation of the parasite (Shadab et al., 2019). Haydock et al. (2015) developed the RNA-Seq-based protocol to exploit the 39 bp SL found at the 5′-end of all *Leishmania* mRNA transcripts (Haydock et al., 2015).

Numerous studies on gene expression profiling were performed in *Leishmania* under various conditions and on its different life stages (McNicoll et al., 2006; Srividya et al., 2007; Guerfali et al., 2008; Lin et al., 2008; Kumari et al., 2008; y Fortéa et al., 2009; Alcolea et al., 2010, 2011; Adaui et al., 2011; Probst et al., 2012; Rabhi et al., 2012, 2013; Beattie et al., 2013). RNA-Seq provided, however, several advantages over other less advanced gene expression profiling approaches such as RT-PCR and microarrays (Nowrousian, 2010). As opposed to hybridization-based methodologies, RNA-Seq can be used without the availability of previous genomic data, which is particularly useful in studying non-model organisms (Vera et al., 2008). It is also less dependent on complete gene annotations as it can identify new genes missed in initial annotations of the genomes of interest (Dillon et al., 2015a). Additionally, RNA-Seq has very low background signal when compared to DNA microarrays having a large dynamic range of expression levels without an upper limit for quantification (Wang et al., 2009). It also provides more accurate quantification of expression levels which could be confirmed through qPCR (Nagalakshmi et al., 2008). Finally, less starting material, total RNA, is required and the results obtained can be highly reproducible for technical and biological replicates (Nagalakshmi et al., 2008; Wang et al., 2009). The most frequently used parameter to measure mRNA abundance based on RNA-Seq data is fragments–or reads-per kilobase of transcript per million mapped reads (FPKM). FPMK represents transcript abundance by considering the RNA length and the total read number in the measurement (Mortazavi et al., 2008). However, other units have been shown to be more effective at contrasting mRNA abundance between samples, including transcripts per millions reads (TPM) (Wagner et al., 2012)

With the vast amount of data generated, there are many opportunities to “translate” this data into forms that can be exploited for drug and vaccine development. The comparison of results between various RNA-seq studies has been hindered by differences in the parasite host, developmental stages of the parasite and experiment lab conditions (for instance, hours of infection) (Dillon et al., 2015a) as such various variables must be addressed and standardized during experimental design to allow for accurate comparative studies of differential gene expression.

Notably, RNA-Seq experiments can also be used to perform metatranscriptomics analyses, reviewed below, at the infection sites and covering the variations in host skin microbiota and insect gut microbiota can provide additional insights into the complex relationships between the bacterial members of the sandfly gut microbiota and the mammalian host skin microbiota in shaping the disease outcome as well as the potential role of LRV present in at least some species of *Leishmania*.

## Sandfly Vector Transcriptomics

Isolating promastigotes from the natural microenvironment (i.e., the vector host) is one potentially useful approach but technically challenging (Alcolea et al., 2016). Previously, *in vitro* infectivity and differential gene expression have been studied in *Leishmania infantum* promastigotes isolated from the stomodeal valve of the sandfly *Phlebotomus perniciosus* (Alcolea et al., 2016). Differential gene expression was determined by RNA shotgun genome microarray hybridization analysis and showed that most differentially expressed genes were involved in regulation of gene expression, intracellular signaling, amino acid metabolism and biosynthesis of surface molecules (Alcolea et al., 2016). Microarray hybridization analysis, however, cannot account for the presence of bacterial or viral genes (Schulze and Downward, 2001). Thus, metatranscriptomics analysis using RNA-Seq on *Leishmania* promastigotes isolated from the inside of the stomodeal valve of the sandfly of the vector could potentially provide more information on the complex relationship existing between parasites, viruses and bacteria co-existing in the vector.

The diversity of the natural gut microbiota of sandflies is acquired from several sources, including feeding on their respective animal and plant sources of blood and sugar, or re-colonization of the gut by the microbes ingested by the terrestrial dwelling larval stages (Dillon et al., 1996; Hillesland et al., 2008; Mukhopadhyay et al., 2012; Peterkova-Koci et al., 2012; Sant’Anna et al., 2012).

A recent 16S rRNA sequence profiling of the gut microbiota from *Lutzomyia longipalpis*, the primary vector of VL in Brazil, revealed 13 distinct bacterial genera (*Bacillus*, *Enterococcus*, *Erwinia*, *Enterobacter*, *Escherichia*, *Klebsiella*, *Lysinibacillus*, *Pseudocitrobacter*, *Providencia*, *Pseudomonas*, *Serratia*, *Staphylococcus*, and *Solibacillus*) (Campolina et al., 2020). Following a co-cultivation of the identified bacteria with various *Leishmania* species in *in vitro* conditions, a growth reduction in all tested parasites was observed suggesting a potential role of the gut microbiota in hindering parasite transmission by the sandfly vector (Campolina et al., 2020). In contrast, the bacterial communities naturally present in the *Phlebotomus duboscqi* sandfly midgut were shown to be essential for the colonization of the midgut by infective stage, metacyclic *Leishmania major* promastigotes (Louradour et al., 2017).

Although insects are hosts to a vast variety of viruses (Telleria et al., 2018), relatively little is known about viruses infecting sandflies (Depaquit et al., 2010). These include phleboviruses such as Toscana viruses (TOSV) (Depaquit et al., 2010). As such, a recent metatranscriptomics analysis of individual mosquitoes unveiled their blood meal sources and uncovered a rich microbial cargo consisting of eukaryotes, prokaryotes, and a high frequency of viral co-infection with 70 known and novel viral species (Batson et al., 2020).

The salivary transcriptome of the *Nyssomyia neivai* sandfly, one of the main vectors of tegumentary leishmaniasis in Brazil, has also been recently performed and highlighted the abundances of several *N. neivai* salivary proteins which can be used as biomarkers of *N. neivai* (Vernal et al., 2020). Thus, looking into the microbial diversity of sandfly vectors gut is of particular interest. Such experiments could also compare and contrast various species of sandfly vectors.

## Findings in *Leishmania* Global Transcriptomics

**FIGURE 3:**
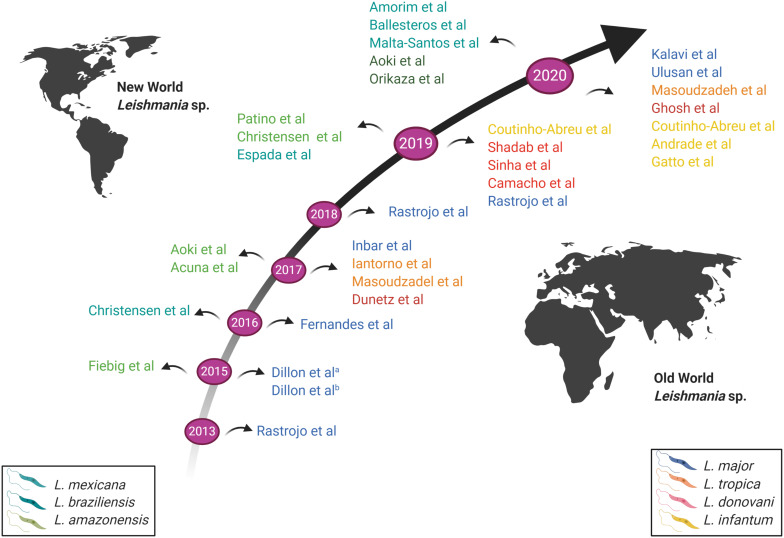
Schematic representation of the time-based evolution of RNA-seq conducted on *Leishmania* parasites. Studies above the arrow represent RNA-Seq conducted on New World *Leishmania* species; studies below the arrow represent RNA-Seq conducted on Old World *Leishmania* species. The various *Leishmania* species studied are indicated by different colors, as shown in the legend. New World *Leishmania* species are shown on the left; Old World *Leishmania* species are shown on the right. Image created with BioRender.com.

The first RNA-Seq global gene expression profiling was performed on *L. major* in 2013 (Rastrojo et al., 2013). A time-based evolution of RNA-Seq experiments conducted on *Leishmania* parasites is presented in Figure 3. Rastrojo et al. (2013) investigated the transcriptome of *L. major* axenic promastigotes and identified a total of 10,285 transcripts including 1,884 novel transcripts that did not match genes previously annotated. They observed and reported the presence of extensive heterogeneity in the SL and polyadenylation addition sites. A sequence comparison of ribosomal protein L23 showed that both genes have identical coding regions, but marked differences both in length and sequence in the 3′-UTRs., which could be linked to the efficiency of the mRNA translation (Rastrojo et al., 2013). Most abundant transcripts included cytosolic heat shock protein 70 (HSP70), various ribosomal proteins, nucleoside transporters, histone H4, peptidases, cyclophilin, *Leishmania*-activated C-kinase antigen (LACK), amastin-like surface protein and alpha tubulin (Rastrojo et al., 2013).

Later Fiebig et al. (2015) used RNA-Seq to characterize and compare the transcriptomes of *L*. *mexicana* promastigotes, axenic and intracellular amastigotes. A comparative analysis of gene expression between promastigotes and amastigotes revealed 3,832 significantly differentially expressed among 9,169 protein-coding genes. Genes associated with the motility of the flagellum were mainly downregulated while those linked to cell surface proteins, transporters, peptidases, and a number of uncharacterized proteins were upregulated in amastigotes. Around 936 novel transcripts were identified providing the first evidence of a link between the whole chromosome duplication event and adaptation to the vertebrate host in this group. Gene ontology-term (GO-term) enrichment analyses exploit the GO system of classification to link genes based on functional characteristics (Bauer et al., 2008). This approach revealed that unfolded protein binding, protein folding, aminoacyl-tRNA ligase activity and tRNA aminoacylation (for protein translation and microtubule motor activity) were preferentially expressed in promastigotes compared to intracellular amastigotes in *L. mexicana*. Genes related to the cytoplasm, calmodulin binding, microtubule-based flagellum, protein folding, and aminoacyl-tRNA ligase activity were preferentially expressed in promastigotes when compared to axenic amastigotes. Chromosome 30 carried genes the expression of which was upregulated in amastigotes suggesting a link between chromosome duplication, gene expression, and parasite host adaptation. Finally, genes having a GO-term annotation related to nucleosome, nucleosome assembly, DNA binding, and DNA replication were preferentially expressed in axenic amastigotes versus intracellular amastigotes (Fiebig et al., 2015).

The differences in gene expression occurring as *Leishmania* acquires its infectivity *in vitro* were also assessed. Dillon et al. (2015a) conducted a global transcriptomic analysis on *L. major* as it gained its infectivity comparing non-infective procyclic promastigotes and infective metacyclic promastigotes. In doing so, they detected 3,138 differentially expressed genes that included iron/zinc transporters, histone H4, adenine amino hydrolysases and 1,044 novel open reading frames (ORFs). Using GO analysis, they revealed that ATP synthesis coupled proton transport and cytoplasm related genes were among the downregulated genes in metacyclic promastigotes. On the other hand, genes encoding for protein kinases and ATP binding were upregulated during differentiation from non-infective procyclic promastigotes and infective metacyclic promastigotes. A widespread alternative *trans*-splicing and polyadenylation was also detected in 8,981 genes (94.2% of a total of 9,530 genes). 5′ and 3′ UTR boundaries for a majority of genes were also identified. No association was found between stage-specific preferential *trans-*splicing or polyadenylation sites and differentially expressed genes. As a result, differences in gene expression levels could not be attributed to stage-regulated alternative mRNA processing (Dillon et al., 2015a).

Furthermore, Iantorno et al. (2017) combined genome-wide high-throughput DNA sequencing with RNA-seq and could identify correlation between chromosome aneuploidy (somy), CNVs, and SNPs across 14 *Leishmania tropica* isolates with total gene expression at the promastigotes stage grown *in vitro*. They found that gene dosage, at the level of individual genes or chromosomal “somy” resulted in more than 85% of total gene expression variation in genes with a two-fold or greater change in expression. Genes encoding membrane-bound transporters were among the most highly upregulated genes. Biopterin transporter BT1 and folate transporter FT1, aquaglyceroporin genes responsible for transport of trivalent antimonials into the cell and the LiMT/LiRos3 transport system, were all previously linked to drug resistance (Marquis et al., 2005; Mandal et al., 2015; Imamura et al., 2016). Notably, in *L. tropica* LRC-L810 recovered from a distinct sandfly vector (*Phlebotomus arabicus* rather than the more commonly known vector *Phlebotomus sergenti*) (Soares et al., 2004) showed a very distinct expression profile (Iantorno et al., 2017). The most highly upregulated gene in isolate LRC-L810 encoded for a receptor-type adenylate cyclase (Iantorno et al., 2017) that has been previously linked to differentiation in African trypanosomes (Fraidenraich et al., 1993), inhibition of the host immune response in *Trypanosoma brucei* (Salmon et al., 2012), and motility in the insect stages (Lopez et al., 2015). *L. tropica* species remains understudied using RNA-seq. As opposed to other *Leishmania* species such as *L. major* (Rastrojo et al., 2013; Dillon et al., 2015a), *L. mexicana* (Fiebig et al., 2015; Patino et al., 2019) and others (Figure 3), very few studies addressed differences in gene expression in *L. tropica* (Iantorno et al., 2017).

Dumetz et al. (2017) also correlated chromosome aneuploidy to changes in gene expression in *Leishmania donovani* during their adaptation to *in vivo* conditions while mimicking natural vertebrate and invertebrate host environments. To mimic the various life stages of the parasite, they used Syrian golden hamsters and sandflies to investigate *in vivo* amastigote and promastigote stages, respectively, and compared the results to *in vitro*-maintained parasites. Aneuploidy was highly variable and correlated with the corresponding transcript levels although aneuploidy-independent regulation of gene expression was also observed. A total of 589 genes were upregulated in promastigotes compared to amastigotes. These mainly belonged to carbon, lipid, and fatty acid metabolism, translation, protein modification, membrane transport, DNA replication and nucleosome assembly, and the function of the flagellum. On the other hand, only 261 genes encoding for amastins and amastin-like proteins, among others were upregulated in amastigotes (Dumetz et al., 2017).

Camacho et al. (2019) also generated the transcriptome of *L. donovani* HU3 promastigotes and identified 2,410 novel transcripts 1,513 of which were homologs to transcripts annotated in *L. major* (Rastrojo et al., 2013). Histone transcripts, alpha tubulin genes, ribosomal proteins, HSP70, and kinetoplastid membrane protein 11 (KMP11) were found to be among the most abundant transcripts (Camacho et al., 2019).

Inbar et al. (2017) described the transcriptome dynamics of *L. major* as it develops inside its natural vector *P. duboscqi* by examining global gene expression in procyclic (PP), nectomonad (NP), and metacyclic promastigotes (MP) compared to BALB/c mice lesion-derived amastigotes (AM) and culture-derived metacyclic promastigotes (CMP). Notably, the greatest number of differentially expressed genes was observed during early transformation from AM to PP in the blood-fed midgut where amastins were down-regulated while multiple cell surface proteins, sugar and amino acid transporters, and genes related to glucose metabolism and cell cycle progression were upregulated. The NP stage revealed changes in genes acting in cell cycle arrest and the upregulation of genes associated with starvation and stress, including autophagic pathways of protein recycling. Maturation to the infective MP showed amastigote-like profiles of surface proteins and metabolism-related genes. The results obtained from comparing gene expression profiles of sandfly derived and culture-derived MP revealed similar results except for an upregulation of transcripts associated with nutrient stress *in vivo* (Inbar et al., 2017).

Coutinho-Abreu et al. (2020b) performed RNA-seq on unaltered midguts of infected *L. longipalpis* sandflies, the vector of *L. infantum*. The sequences obtained from procyclic, nectomonad, leptomonad or metacyclic promastigote stages were grouped into distinct populations based on principal component analysis, with the procyclic stage being the most distinct. A total of 836 genes were differentially expressed between procyclic and nectomonad promastigotes, 113 between nectomonad and leptomonad promastigotes and 302 between leptomonad and metacyclic promastigotes. Most of the differentially expressed genes were uniquely expressed in each stage and not in other stages. Stage-specific markers included genes encoding a zinc transporter in procyclics, a beta-fructofuranidase in nectomonads, a surface antigen-like protein in leptomonads, and an amastin-like surface protein in metacyclics (Coutinho-Abreu et al., 2020b). Conversely, the presence of *L. infantum* in *L. longipalpis* sandflies produced a limited change in the sandfly’s transcript expression profile (Coutinho-Abreu et al., 2020a). As such, the parasite appeared to modulate gene expression early on in the developmental cycle (Days 1 to 4) in order to overcome the barriers imposed by the midgut of the insect, then most of the differentially expressed transcripts were up-regulated with small fold changes at later time points (Day 6 and onward) with only slight changes observed in midgut gene expression (Coutinho-Abreu et al., 2020a). The most abundant transcripts in promastigotes and amastigotes of various *Leishmania* species are summarized in Tables 1, 2, respectively.

**TABLE 1:** Top twenty most abundant transcripts in promastigotes of various *Leishmania* species.

Reference	Rastrojo et al. (2013) ^*a*^		Dillon et al. (2015b) ^*b*^		Fiebig et al. (2015) ^*c*^		Dumetz et al. (2017) ^*d*^	
Species	*L. major*		*L. major*		*L. mexicana*		*L. donovani*	
#	Gene ID	Product description	Gene ID	Product description	Gene ID	Product description	Gene ID	Product description
1	LmjF.28.T2770	HSP70–II	LmjF.12.0940	PSA2**	LmxM.01.0410	–	LdBPK_230 013000	PAP2 superfamily, putative
2	LmjF.35.T0240	ribosomal protein L30	LmjF.12.1000	PSA2	LmxM.03.0250	ribosomal protein L38, putative	LdBPK_040 015700	COPI associated protein, putative
3	LmjF.28.T2780	HSP70–II	LmjF.12.0980	PSA2	LmxM.03.0430	60S acidic ribosomal protein P2, putative	LdBPK_350 038000	cystathione gammalyase, putative
4	LmjF.36.T1940	inosine–guanosine transporter (NT2)	LmjF.34.3645	hypothetical protein (pseudogene)	LmxM.04.0750	60S ribosomal protein L10, putative	LdBPK_310 032500	3′–nucleotidase/nuclease precursor, putative
5	LmjF.31.T0900	hypothetical protein	LmjF.12.0910	PSA	LmxM.06.0010	histone H4	LdBPK_020 011100	hypothetical protein, conserved
6	LmjF.28.T2205	ribosomal protein S29	LmjF.20.0150	hypothetical protein	LmxM.06.0570	60S ribosomal protein L23a, putative	LdBPK_360 045100	EF–hand domain pair, putative
7	LmjF.35.T2220	KMP11*	LmjF.12.0920	PSA	LmxM.07.0680	40S ribosomal protein S9, putative	LdBPK_340 015900	amastin–like protein
8	LmjF.19.T0983	–	LmjF.12.1040	surface antigen protein putative	LmxM.08_29.2461	60S ribosomal protein L13, putative	LdBPK_120 012700	–
9	LmjF.35.T0600	ribosomal protein L18a	LmjF.12.1020	surface antigen protein putative	LmxM.09.1340	histone H2B	LdBPK_120 014800	PSA2
10	LmjF.06.T0010	histone H4	LmjF.14.1360	inositol-3-phosphate synthase (INO1)	LmxM.10.0070	ribosomal protein l35a, putative	LdBPK_260 017500	HSP70
11	LmjF.35.T3800	ribosomal protein L23	LmjF.12.0860	surface antigen protein putative	LmxM.11.0970	40S ribosomal protein S5	LdBPK_180 021700	hypothetical protein
12	LmjF.36.T3620	hypothetical protein, conserved	LmjF.12.1060	surface antigen protein putative	LmxM.13_158814	novel transcript	LdBPK_120 013500	PSA2
13	LmjF.35.T2210	KMP11*	LmjF.07.0745	hypothetical protein	LmxM.13.0280	alpha tubulin	LdBPK_200 018100	small myristoylated protein-1
14	LmjF.28.T2460	ribosomal protein S29	LmjF.12.0780	surface antigen protein 2 precursor	LmxM.13.0560	60S ribosomal protein L18, putative	LdBPK_190 014100	ATG8/AUT7/APG8/PAZ2,putative
15	LmjF.20.T1285	–	LmjF.28.1570	hydrolase alpha/beta fold family putative	LmxM.13.1670	60S ribosomal protein L44, putative	LdBPK_190 020100	hypothetical protein
16	LmjF.31.T0964	–	LmjF.32.2260	HSP20	LmxM.14.1270	ubiquitin/ribosomal protein S27a, putative	LdBPK_030 011600	Uroporphyrinogen-III synthase HemD, putative
17	LmjF.31.T0895	–	LmjF.26.0640	10 kDa heat shock protein putative	LmxM.15_455510	novel transcript	LdBPK_360 057700	paraflagellar rod component, putative
18	LmjF.35.T3290	ribosomal protein L31	LmjF.07.0745	hypothetical protein	LmxM.15.0010	histone H4	LdBPK_360 076600	tartrate-sensitive acid phosphatase acp-3.2, putative
19	LmjF.13.T0570	ribosomal protein S12	LmjF.36.4050	hypothetical protein	LmxM.15.1160	tryparedoxin peroxidase	LdBPK_250 026700	2,4-dihydroxyhept-2-ene-1,7-dioicacidaldolase, putative
20	LmjF.35.T3790	ribosomal protein L23	LmjF.31.1440	hypothetical protein	LmxM.15.1240	nucleoside transporter 1, putative	LdBPK_310 040800	iron/zinc transporter protein-like protein

*Kinetoplastid membrane protein-11; **Promastigote surface antigen protein 2.

*^a^In Rastrojo et al. (2013), promastigotes were grown to mid log phase by seeding cultures at 1 × 10^6^ cells/mL, and collected for RNA isolation when the culture density reached 6.1 × 10^6^ cells/mL (mid-logarithmic phase of growth).*

*^b^In Dillon et al. (2015b), murine macrophages from C57BL/6 mice were infected with L. major metacyclic promastigotes at a ratio of 5 parasites per macrophage for 4 h. L. major promastigotes were not split for more than 5 passages. List of upregulated genes was obtained by comparing metacyclic promastigotes to amastigotes at 4 hpi.*

*^c^In Fiebig et al. (2015), promastigotes were harvested in late exponential growth phase (around 1 × 10^7^ cells/ml) and collected for RNA isolation.*

*^d^In Dumetz et al. (2017), promastigotes were harvested in logarithmic phase (day 4). List of upregulated genes was obtained by comparing differential expression in metacyclic promastigotes to amastigotes.*

**TABLE 2:** Top twenty most abundant transcripts in amastigotes of three major *Leishmania* species.

Reference	Dillon et al. (2015b) ^*a*^		Fiebig et al. (2015) ^*b*^		Dumetz et al. (2017) ^*c*^	
Species	*L. major*		*L. mexicana*		*L. donovani*	
#	Gene ID	Product description	Gene ID	Product description	Gene ID	Product description
1	LmjF.12.1060	surface antigen protein putative	LmxM.01.0410	unspecified product	LdBPK_260011800	Thioredoxin, putative
2	LmjF.12.1020	surface antigen protein putative	LmxM.03.0250	ribosomal protein L38, putative	LdBPK_090017600	DNA-directed RNApolymerase III subunit, putative
3	LmjF.12.0780	surface antigen protein 2 precursor	LmxM.03.0430	60S acidic ribosomal protein P2, putative	LdBPK_350057300	hypothetical protein
4	LmjF.12.0920	PSA	LmxM.04.0750	60S ribosomal protein L10, putative	LdBPK_190015500	FYVE zinc finger containing protein, putative
5	LmjF.12.1040	surface antigen protein putative	LmxM.06.0010	histone H4	LdBPK_300018500	hypothetical protein
6	LmjF.12.0910	PSA	LmxM.06.0570	60S ribosomal protein L23a, putative	LdBPK_160011500	RNA recognition motif
7	LmjF.12.0810	PSA2	LmxM.06.0580	60S ribosomal protein L23a, putative	LdBPK_340024100	Amastin surface glycoprotein, putative
8	LmjF.12.0860	surface antigen protein putative	LmxM.07.0680	40S ribosomal protein S9, putative	LdBPK_290023800	MutS-like protein
9	LmjF.12.0940	PSA2	LmxM.08_29.1090	ribosomal protein L1a, putative	LdBPK_200019600	hypothetical protein
10	LmjF.12.0890	surface antigen protein 2 putative	LmxM.08_29.1740	histone H2A, putative	LdBPK_350041100	Pre-rRNA-processing protein PNO1, putative
11	LmjF.12.1000	PSA2	LmxM.08_29.1800	40S ribosomal protein S15A, putative	LdBPK_320045600	hypothetical protein
12	LmjF.12.0980	PSA2	LmxM.08_29.2461	60S ribosomal protein L13, putative	LdBPK_160010000	hypothetical protein
13	LmjF.12.0960	surface antigen protein 2 putative	LmxM.08.1030	unspecified product	LdBPK_110016600	hypothetical protein
14	LmjF.04.0040	hypothetical protein	LmxM.08.1030a	unspecified product	LdBPK_300028700	Zinc finger
15	LmjF.25.1120	aldehyde dehydrogenase mitochondrial precursor (ALDH2)	LmxM.08.1070	cathepsin L-like protease, putative	LdBPK_300010200	hypothetical protein
16	LmjF.26.1340	DNA ligase k alpha putative	LmxM.09.1340	histone H2B	LdBPK_040017600	casein kinase I, putative
17	LmjF.12.0830	surface antigen protein 2 putative	LmxM.10.0070	ribosomal protein l35a, putative	LdBPK_310015600	KIAA1430 homolog, putative
18	LmjF.34.2940	hypothetical protein conserved	LmxM.13.0280	alpha tubulin	LdBPK_110016700	hypothetical protein
19	LmjF.24.1280	amastin-like surface protein-like protein	LmxM.13.0450	hypothetical protein, conserved	LdBPK_230018900	5-formyltetrahydrofolatecyclo-ligase family, putative
20	LmjF.27.1080	hypothetical protein conserved	LmxM.13.1670	60S ribosomal protein L44, putative	LdBPK_210015500	hypothetical protein

*^a^In Dillon et al. (2015b), murine macrophages from C57BL/6 mice were infected with L. major metacyclic promastigotes at a ratio of 5 parasites per macrophage for 4 h. L. major promastigotes were not split for more than 5 passages. List of upregulated genes was obtained by comparing amastigotes at 4 hpi vs. 24 hpi. ^b^In Fiebig et al. (2015), to generate intracellular amastigotes, promastigotes were left to grow into stationary phase (from 1 × 10^5^ to 2 × 10^7^ cells/ml) and then incubated with mouse macrophages at a parasite to macrophage ratio of 20 to 1. Leishmania-infected macrophages were harvested 24 h post-infection and collected for RNA isolation. ^c^In Dumetz et al. (2017), amastigotes were recovered from infected hamsters and resuspended at 2 × 10^8^ amastigotes per ml in phosphate-buffered saline (PBS) for RNA extraction. List of upregulated genes was obtained by comparing differential expression in amastigotes to metacyclic promastigotes.*

## Host Cell’s Response to Infection

Another advantage of using RNA-Seq is to be able to look into gene expression changes in the host cell following parasitic infections in so called dual RNA-Seq experiments (Dillon et al., 2015b; Fernandes et al., 2016). Dillon et al. (2015b) aimed at identifying global changes in gene expression using murine macrophages from C57BL/6 mice and *L. major* at 4, 24, 48, and 72 h post-infection (hpi). The most significant response to infection by the macrophage was observed at 4 hpi, with 6,897 mouse genes significantly differentially expressed between uninfected and infected cells. Genes related to both pro- and anti-inflammatory immune responses and glycolysis were substantially upregulated, and genes related to lipid metabolism, biogenesis, and Fc gamma receptor-mediated phagocytosis were downregulated in the murine macrophages. Genes linked to the mitigation of oxidative stress by the host immune system were upregulated while genes related to translation, cell signaling, fatty acid biosynthesis, and flagellum structure were downregulated in *L. major* amastigotes. KEGG enrichment analysis revealed that the differentially expressed genes in macrophages were related to immune response (cytokine-cytokine receptor interactions and arginine and proline metabolism) and glycolysis. The immunoregulatory activity detected in macrophages infected with *L. major* has been previously also observed in macrophages stimulated with lipopolysaccharide (LPS) (Fleming et al., 2015). Dillon et al. (2015b) also observed a total of 2,962 genes that were differentially expressed in metacyclic promastigotes compared to amastigotes. These were mostly thought, based on their annotation, to be involved in reducing the effects of an oxidative stress response exerted on the parasite by the innate immune response taking place in the phagosome (Zhang et al., 2010). Heat shock proteins, especially HSP83, multiple tryparedoxin peroxidase family members, and multiple cyclophilins were all upregulated upon entry of metacyclic promastigotes into host cells (Dillon et al., 2015b). Human monocytes infected with *L. major* also revealed an upregulation of pro-inflammatory cytokine and cytokines receptors including IL1A, IL1RN, IL6, and IL6R (Kalavi et al., 2020).

It’s noteworthy that Fernandes et al. (2016) used a dual transcriptome approach to profile gene expression in human CD14^+^ monocytes infected by *L. major* and *Leishmania amazonensis* at different time points (4, 24, 48, and 72 hpi). A temporal expression pattern was observed in both the macrophage and the parasite as macrophage response and parasite transformation stabilized shortly (4 hpi) after entry of the *Leishmania* into the host. No significant difference was observed in the parasite species transcriptomes or the macrophage response between the two different *Leishmania* species. Following infection with *L. major*, 5,713 human genes were differentially expressed between uninfected and infected macrophages. Similar results were obtained with *L. amazonensis*, mainly detecting genes encoding for inflammatory cytokines (IL-1β, TNF, TNF superfamily members, and IL-6) and a number of immunomodulators. Immunomodulators included: prostaglandin-endoperoxide synthase 2 (PTGS2), colony-stimulating factors 1 and 2 (CSF1 and CSF2), and superoxide dismutase 2 (SOD2). SOD2 was upregulated only following infection with *L. major*. Of interest were the host metallothionein 1 family members with their role in the disease outcome requiring further attention. On the parasite side, genes encoding for amastin, *gp63*, kinesin, flagellar attachment zone protein, AAT family members, dynein and cysteine peptidase B were all upregulated post-infection in both *Leishmania* species (Fernandes et al., 2016).

Moreover, Shadab et al. (2019) investigated host-specific and parasite-specific factors modulating the host-parasite interaction in *L. donovani* AG83 strain. They infected murine peritoneal macrophages with either the virulent or a non-virulent variant of the parasite derived from the AG83 strain. Infection with virulent *L. donovani* strains revealed suppression of many important cellular processes, including protein synthesis. Genes encoding virulence factors and those important for parasite survival were significantly upregulated in the intracellular virulent amastigotes. In contrast, genes involved in the immune stimulations and negative regulation of the cell cycle and transcriptional regulation were also all upregulated in the non-virulent strains. The non-virulent *L. donovani* AG83 strain was generated by Sinha et al. (2018) through continuous *in vitro* passages and revealed global changes in the genome and transcriptome of the serially passaged *L. donovani* AG83 promastigotes. No massive transcript expression changes between the virulent and non-virulent AG83 promastigotes were observed and among the few differentially expressed genes were the one coding for acid phosphatase playing a role in virulence and endosome sorting (Katakura and Kobayashi, 1988), and cyclin-dependent kinase pho85-like protein. Homologs of the latter gene was previously implicated environmental signaling in yeast in response to stressful environmental conditions (Carroll and O’Shea, 2002).

Dual RNA sequencing of both enucleated fibroblasts (cytoplasts) and intracellular *L. amazonesis* has been recently performed to gain further insights in the parasite’s control over the host cell (Orikaza et al., 2020). This experimental system demonstrated that the parasite multiplication and biogenesis of large parasitophorous vacuoles were independent of the host cell nucleus (Orikaza et al., 2020). Infected cytoplast transcripts were enriched in phagolysosome-related pathway, pro-survival, and SerpinB-mediated immunomodulation compared to control non-infected cytoplasts. Notably, these results suggested that a parasite-mediated control of host cell transcripts half-life was beneficial to the parasite’s intracellular multiplication and evasion of the host immune response (Orikaza et al., 2020).

*L*. *infantum* did not activate the inflammasomes in the THP-1 human macrophage infected cells (Gatto et al., 2020). In fact, *L*. *infantum* triggered a gene expression pattern more similar to non-infected THP-1 cells yet very different from LPS-stimulated cells. Some of the most up-regulated genes in *L*. *infantum*-infected cells were related to: cell cycle (CDC20, CDKN2C, CNNL2), glycolysis/gluconeogenesis (ENO1), metabolism of carbohydrates (AGRN), signal transduction (RIT1, RPS6KA1, ILR6, SFPQ), MAPK signaling (RPS6KA1), gene expression (NOTCH2), ubiquitin mediated proteolysis (CDC20) and interleukin signaling (TNFRSF14, TNFRSF1B, ILR6, CSF1). Genes associated with the inflammasome signaling pathway were not differentially expressed and caspase-1 activation and IL-1β production were absent following infection (Gatto et al., 2020). Genes significantly upregulated or downregulated in macrophages following infection with various *Leishmania* species are summarized in Tables 3, 4, respectively.

**TABLE 3:** Top twenty upregulated genes in macrophages following infection with various *Leishmania* species.

Reference	Dillon et al. (2015b) ^*a*^		Fernandes et al. (2016) ^*b*^		Shadab et al. (2019) ^*c*^	
	
Cell Types	C57BL/6 mice peritoneal macrophages		Human macrophages		C57BL/6 mice peritoneal macrophages	
	
Species	*L. major*		*L. major*		*L. donovani*	
	
#	ID	Product description	ID	Product description	MGI ID	Product description
1	ENSMUSG00000031762	metallothionein 2	ENSG00000168334	xin actin-binding repeat containing 1	MGI:1340899	chitinase-like 1
2	ENSMUSG00000031765	metallothionein 1	ENSG00000122641	inhibin beta A	MGI:2685490	transmembrane protein 132E
3	ENSMUSG00000010051	hyaluronoglucosaminidase 1	ENSG00000164400	colony stimulating factor 2 (granulocyte-macrophage)	MGI:2135946; MGI:2443796	CD163 antigen; CD163 molecule -like 1
4	ENSMUSG00000045362	tumor necrosis factor receptor superfamily member 26	ENSG00000010310	gastric inhibitory polypeptide receptor	MGI:2676631	interleukin 17F
5	ENSMUSG00000050914	ankyrin repeat domain 37	ENSG00000125144	metallothionein 1G	MGI:107364; MGI:2676631	interleukin 17A; interleukin 17F
6	ENSMUSG00000037709	family with sequence similarity 13 member A	ENSG00000205364	metallothionein 1M	MGI:96062; MGI:2685715	histidine decarboxylase; hdc homolog, cell cycle regulator
7	ENSMUSG00000000794	potassium intermediate/small conductance calcium-activated channel subfamily N member 3	ENSG00000169908	transmembrane 4 L six family member 1	MGI:2146080	shisa family member 8
8	ENSMUSG00000031444	coagulation factor X	ENSG00000169715	metallothionein 1E	MGI:1352744; MGI:2672905	deltex 1, E3 ubiquitin ligase; deltex 4, E3 ubiquitin ligase
9	ENSMUSG00000089929	B cell leukemia/lymphoma 2 related protein A1b	ENSG00000115009	chemokine (C-C motif) ligand 20	MGI:1916978	caspase recruitment domain family, member 11
10	ENSMUSG00000078566	BCL2/adenovirus E1B interacting protein 3	ENSG00000073861	T-box 21	MGI:1858224	carbohydrate (chondroitin 6/keratan) sulfotransferase 3
11	ENSMUSG00000029321	solute carrier family 10 (sodium/bile acid cotransporter family) member 6	ENSG00000181773	G protein-coupled receptor 3	MGI:1306780	early growth response 3
12	ENSMUSG00000025161	solute carrier family 16 (monocarboxylic acid transporters) member 3	ENSG00000149635	osteoclast stimulatory transmembrane protein	MGI:1917066	marginal zone B and B1 cell-specific protein 1
13	ENSMUSG00000062345	serine (or cysteine) peptidase inhibitor clade B member 2	ENSG00000125084	wingless-type MMTV integration site family member 1	MGI:105086	POU domain, class 2, associating factor 1
14	ENSMUSG00000031709	TBC1 domain family member 9	ENSG00000166670	matrix metallopeptidase 10 (stromelysin 2)	MGI:101774	CD79A antigen (immunoglobulin- associated alpha)
15	ENSMUSG00000035105	egl-9 family hypoxia-inducible factor 3	ENSG00000128342	leukemia inhibitory factor	MGI:96543; MGI:96547	interleukin 1 beta; interleukin 1 receptor antagonist
16	ENSMUSG00000024730	membrane-spanning 4-domains subfamily A member 8A	ENSG00000117090	signaling lymphocytic activation molecule family member 1	MGI:1919299	tumor necrosis factor receptor superfamily, member 13c
17	ENSMUSG00000039753	F-box and leucine-rich repeat protein 5	ENSG00000205358	metallothionein 1H	MGI:88319	CD19 antigen
18	ENSMUSG00000056054	S100 calcium binding protein A8 (calgranulin A)	ENSG00000166923	gremlin 1 DAN family BMP antagonist	MGI:2443630	sialic acid binding Ig-like lectin G
19	ENSMUSG00000024679	membrane-spanning 4-domains subfamily A member 6D	ENSG00000129521	egl-9 family hypoxia-inducible factor 3	MGI:2138647	Fc receptor-like A
20	ENSMUSG00000019916	procollagen-proline 2-oxoglutarate 4-dioxygenase (proline 4-hydroxylase) alpha 1 polypeptide	ENSG00000164181	ELOVL fatty acid elongase 7	MGI:96941	chymase 1, mast cell

*^a^In Dillon et al. (2015b), peritoneal macrophages were isolated from C57BL/6 mice and infected with L. major metacyclic promastigotes at a ratio of 5 parasites per macrophage. The list of up-regulated genes shows top genes consistently upregulated across all timepoints upon infection by L. major at 4, 24, 48, and 72 hpi. ^b^In Fernandes et al. (2016), human macrophages were derived from purified monocytes and infected using a ratio of 5 L. major parasites per macrophage for 4 h. List of upregulated genes was obtained by comparing differential gene expression in L. major-infected human macrophages relative to uninfected controls at 4 hpi. ^c^In Shadab et al. (2019), peritoneal C57BL/6 mice macrophages were infected at a parasite to macrophage ratio of 10:1 for 12 h. List of top upregulated genes was obtained by comparing differential gene expression in L. major-infected human macrophages relative to uninfected controls.*

**TABLE 4:** Top twenty down-regulated genes in macrophages following infection with various *Leishmania* species.

Reference	Dillon et al. (2015a) ^*a*^		Fernandes et al. (2016) ^*b*^		Shadab et al. (2019) ^*c*^	
Cell Types	57BL/6 mice peritoneal macrophages		Human macrophages		57BL/6 mice peritoneal macrophages	
Species	*L. major*		*L. major*		*L. donovani*	
#	ID	Product description	ID	Product description	MGI ID	Product description
1	ENSMUSG0000 0013236	protein tyrosine phosphatase receptor type S	ENSG00000121933	adenosine A3 receptor	MGI:102709	caveolin 1, caveolae protein
2	ENSMUSG0000 0036067	solute carrier family 2 (facilitated glucose transporter) member 6	ENSG00000220008	leucine rich repeat and Ig domain containing 3	MGI:2136773	CWC22 spliceosome-associated protein
3	ENSMUSG0000 0029581	fascin homolog 1 actin bundling protein	ENSG00000127533	coagulation factor II (thrombin) receptor-like 3	MGI:2145242	arrestin domain containing 3
4	ENSMUSG0000 0021451	sema domain immunoglobulin domain (Ig) transmembrane domain (TM) and short cytoplasmic domain (semaphorin) 4D	ENSG00000150681	regulator of G-protein signaling 18	MGI:107422	heat shock protein 4 like
5	ENSMUSG0000 0053063	C-type lectin domain family 12 member a	ENSG00000179144	GTPase IMAP family member 7	MGI:88590	cytochrome P450, family 1, subfamily b, polypeptide 1
6	ENSMUSG0000 0076431	SRY (sex determining region Y)-box 4	ENSG00000137834	SMAD family member 6	MGI:2449975	IQ motif containing GTPase activating protein 2
7	ENSMUSG0000 0091747	–	ENSG00000245848	CCAAT/enhancer binding protein (C/EBP) alpha	MGI:2443069	peptidylprolyl isomerase domain and WD repeat containing 1
8	ENSMUSG0000 0025574	thymidine kinase 1	ENSG00000257108	NHL repeat containing 4	MGI:96969; MGI:1913910; MGI:1915147	met proto-oncogene; SAFB-like, transcription modulator; RNA (guanine-7-) methyltransferase
9	ENSMUSG0000 0002602	AXL receptor tyrosine kinase	ENSG00000107719	phosphatase domain containing paladin 1	MGI:2443342	post-GPI attachment to proteins 1
10			ENSG00000180340	frizzled class receptor 2	MGI:1095419	lysine (K)-specific demethylase 6A
11			ENSG00000152804	hematopoietically expressed homeobox	MGI:2183747	FYVE, RhoGEF, and PH domain containing 4
12			ENSG00000136457	chondroadherin	MGI:107483; MGI:108427; MGI:1924705	ral guanine nucleotide dissociation stimulator-like 2; insulin-like 3; rearranged L-myc fusion sequence
13			ENSG00000234432	–	MGI:1196332	Rho GTPase activating protein 6
14			ENSG00000134222	proline/serine-rich coiled-coil 1	MGI:1914829	cytoplasmic polyadenylation element binding protein 4
15			ENSG00000163606	CD200 receptor 1	MGI:1931053	membrane-associated ring finger (C3HC4) 7
16			ENSG00000004799	pyruvate dehydrogenase kinase isozyme 4	MGI:107448	lysosomal trafficking regulator
17			ENSG00000177599	zinc finger protein 491	MGI:1923036	cytoskeleton associated protein 5
18			ENSG00000204131	NHS-like 2	MGI:1913975	leucine-rich repeat kinase 2
19			ENSG00000196934	NA	MGI:96562	interleukin 7 receptor
20			ENSG00000213203	GTPase IMAP family member 1	MGI:1914769	DENN/MADD domain containing 4C

*^a^In Dillon et al. (2015b), peritoneal macrophages were isolated from C57BL/6 mice and infected with L. major metacyclic promastigotes at a ratio of 5 parasites per macrophage. The list consists of nine down-regulated genes consistently downregulated across all timepoints upon infection by L. major at 4, 24, 48, and 72 hpi. ^b^In Fernandes et al. (2016), human macrophages were derived from purified monocytes and infected using a ratio of 5 L. major parasites per macrophage for 4 h. List of down-regulated genes was obtained by comparing differential gene expression in L. major-infected human macrophages relative to uninfected controls at 4 hpi. ^c^In Shadab et al. (2019), peritoneal C57BL/6 mice macrophages were infected at a parasite to macrophage ratio of 10:1 for 12 h. List of top down-regulated genes was obtained by comparing differential gene expression in L. major-infected human macrophages relative to uninfected controls.*

## Assessment of Metabolic Pathways

RNA-Seq was also used to assess the importance of L-arginine pathway in *L. amazonensis* promastigotes and axenic amastigotes (Aoki et al., 2017). The L-arginine pathway in *Leishmania* is important during the parasite life cycle and infection of the mammalian macrophages (Cunningham, 2002). Nitric oxide (NO) production is a defense mechanism used by macrophages being produced by nitric oxide synthase 2 (NOS2) in the presence of the amino acid L-arginine as substrate (Cunningham, 2002). Arginase on the other hand, reduces NO production by limiting the availability of L-arginine and favoring the survival of *Leishmania* in the macrophage (Camargo et al., 1978; Aoki et al., 2020). Aoki et al. (2017) studied gene expression differences between *L*. *amazonensis* wild-type (*La*-WT) and *L*. *amazonensis* arginase knockout (*La*-arg^–^) promastigotes and axenic amastigotes. In doing so, they identified a total of 8,253 transcripts in both strains of which 60% encoded hypothetical proteins, 443 were novel transcripts that did not match any previously annotated genes, and 85% were constitutively expressed. WT amastigotes had lower levels of arginase and higher levels of glutamate-5-kinase compared to WT promastigotes. *La*-arg^–^ promastigotes had increased levels of pyrroline 5-carboxylate reductase, but decreased levels of arginosuccinate synthase, pyrroline 5-carboxylate dehydrogenase, acetylornithine deacetylase and spermidine synthase compared to the WT. Thus, arginase activity is important in *Leishmania* gene expression modulation during its differentiation and adaptation to environmental changes. Acuña et al. (2017) also described an arginase-dependent NOS-like activity in *L*. *amazonensis* which could be important to trigger parasite differentiation during infection of macrophages. They suggested that NO production could be arginase-dependent and higher levels of NO were produced in axenic amastigotes compared to promastigotes. Aoki et al. (2019) further expanded the study of the role of arginase in the modulation of virulence factors involved in parasite recognition, growth and differentiation in *L. amazonensis*. Arginase was found to upregulate membrane markers that affect parasite recognition, autophagy-related genes implicated in parasite differentiation and amastins that impact amastigote replication and survival, while modulating oxidative-stress related genes (Aoki et al., 2019). The parasite’s arginase activity also appeared to modulate gene expression in the host macrophage including in the immune response and amino acid transport and metabolism (Aoki et al., 2020).

Recently, Malta-Santos et al. (2020) investigated the importance of the polyamine biosynthetic pathway in CL lesions. Diffuse CL was associated with higher concentrations of amino acids, polyamines and its substrate transporters compared to MCL or localized CL. The most upregulated transcripts in diffuse CL lesions were CAT2A (isoform encoded by SLC7A2), ARG1 and SMS compared to MCL lesions acting in the regulation of arginine availability. A differential gene expression of polyamine metabolism-related genes of patients’ lesions was associated with parasite loads and the leishmaniasis disease outcome (Malta-Santos et al., 2020).

## Direct Application to Skin Biopsies

RNA-Seq can also be directly applied on human tissues such as skin biopsies. Christensen et al. (2016, 2019) studied gene expression changes in the host and the parasite using skin biopsies from *Leishmania braziliensis-*infected patients with, respectively, localized cutaneous leishmaniasis (LCL) and diffuse cutaneous leishmaniasis (DCL). In Christensen et al. (2016), detectable parasite transcripts in only one group of patients were found in which an increase in B lymphocyte-specific and immunoglobulin transcripts in the lesions, and an upregulation of immune inhibitory molecules was observed. Patients negative for parasite transcripts had a decrease in B cell activation, but increased expression of antimicrobial genes and genes encoding skin barrier functions, and which were different from transcripts expressed in human macrophages *in vitro.* Therefore, the differences in gene expression observed *in vitro* in macrophages does not accurately reflect the one observed from skin lesions, as expected as in the skin biopsies one collects multiple cells. Hence it is not possible/very difficult to compare the transcriptome of macrophages in cell culture versus mix of cells, including macrophages, from a biopsy. Highly expressed genes in the parasite included those encoding for cysteine peptidase and synthase, glycerophosphoryl phosphodiesterase family protein and transport protein SEC13 among others. Upregulated genes in lesions with detectable parasite transcripts encoded immunoglobulin fragments, CXCL8 (encoding chemokine IL-8, granulocyte and neutrophil chemotaxis), IL-21 (B cell proliferation), and granulysin (cellular cytotoxicity) encoding genes (Christensen et al., 2016).

Skin lesions from BALB/c mice infected with *L. major* showed an upregulation of FCGR4, CCL4, CXCL9, Arg1, and IL-1β and an enrichment of the Triggering Receptor Expressed on Myeloid Cells 1 (TREM1) signaling pathway (Ulusan et al., 2020).

To date, very few metatranscriptomics studies have been performed on *L. tropica*. These included RNA-Seq performed on skin biopsies in *L. tropica* infected patients (Masoudzadeh et al., 2017). Unfortunately, the usefulness and application of this study for downstream comparative analysis remains limited due to the unavailability of reads from *L. tropica* which were not analyzed nor deposited in a public database. Notably, transcription profiling of *L. tropica*-infected skin identified over 5,000 differentially regulated human genes compared to uninfected controls (Masoudzadeh et al., 2017). Gene ontology enrichment analysis indicated that upregulated genes were mostly involved in immune response activation, extracellular matrix degradation, inflammatory cell recruitment, cytotoxicity and antimicrobial peptides (Masoudzadeh et al., 2017). Similarly to the previous report by Christensen et al. (2016), they observed an increase in B lymphocyte-specific (immunoglobulin lambda-like polypeptide 5, IGLL5) and immunoglobulin receptor (Fc Fragment of IgG Receptor Ia, FCGR1A) transcripts. These findings highlight the role of B cells at the infection site. Downregulated genes on the other hand, encoded mainly for structural proteins associated with muscle contraction (e.g., myosin 2, alpha 1 actin, myosin 1, and troponin C type 2) (Masoudzadeh et al., 2017). Furthermore, shared and unique functional transcriptional pathways in *L. tropica* infected skin lesions of ulcerative CL (UCL) and non-ulcerative CL (NUCL) patients were investigated using RNA-Seq (Masoudzadeh et al., 2020a). Inflammatory cytokines and chemokines were differentially expressed in the UCL and NUCL lesions. Transcriptional pathways for Fcγ receptor dependent phagocytosis were enriched in both conditions (Masoudzadeh et al., 2020a).

Notably, Christensen et al. (2019) using meta-transcriptomic analysis of biopsies from patients with DCL, demonstrated an infiltration of atypical B cells producing a dominance of the IgG4 isotype in these *L. amazonensis* infected patients. They additionally, revealed the absence of cytotoxic and T_*H*_2 cell responses. A regulatory phenotype was observed in macrophages with some genes being actively expressed such as an ATP-binding cassette, subfamily B, member 5 (ABCB5), dendritic cell specific transmembrane protein (DC-STAMP), and a secreted phosphoprotein 1 (SPP1) among others. Gene expression in DCL lesions resembled patterns obtained from *in vitro* parasite growth in resting macrophages. Furthermore, 336 genes in *L. amazonensis* were upregulated in LCL compared to DCL. Among these upregulated genes, seven potential virulence factors and four stress response genes were detected, including GP63, proteins containing leucine-rich repeats, a cyclophilin protein, a protein containing a RmlC-like jelly roll fold domain, and a Betv1-like superfamily protein.

The blood of *L. braziliensis* infected patients was also recently analyzed using RNA-seq to determine systemic responses that might be influencing the disease (Amorim et al., 2020). A strong interferon stimulated gene (ISG) signature has been identified which was correlated with an increase in circulating monocytes and macrophages (Amorim et al., 2020). A cytotoxicity signature was correlated with an increase in cytolytic cells (Amorim et al., 2020).

Similarly, the blood of VL-HIV patients was studied through transcriptional profiling (Adriaensen et al., 2020). A downregulation of genes associated with host cellular activity and immunity, and upregulation of antimicrobial peptide activity in phagolysosomes was observed in patients that were successfully treated with AmBisome or AmBisome/miltefosine, in contrast, no pathway enrichment among differentially expressed genes was observed in treatment failure patients (Adriaensen et al., 2020).

## Assessing Drug Resistance

The appearance of drug resistance phenotypes is a major concern hindering available anti-leishmanial treatment (Sundar and Singh, 2016). Rastrojo et al. (2018) identified genomic and transcriptomic alterations associated with experimental resistance in *L. donovani*, *L. infantum* and *L. major* promastigotes and amastigotes in response to common drugs used against VL including: trivalent antimony (S line), amphotericin B (A line), miltefosine (M line), and paromomycin (P line). In total, 1,006 differentially expressed transcripts were identified in the S line, 379 in the A line, 146 in the M line, and 129 in the P line. Changes in chromosomal aneuploidy and amplification/deletion of particular regions were correlated with resistance. A series of genes were identified as possible drivers of the resistance phenotype. The S line included peptidyl dipeptidase, amastin, and amastin-like surface proteins among others. The M line included protein kinase, carboxypeptidase and amino acid transporter AAT1.4 among others. The P line included flavoprotein subunit-like protein, amastin-like protein, protein associated with differentiation and iron/zinc transporter protein-like protein (LIT1) among others. Finally, the A line included a sterol 24-c- methyltransferase and phosphatidylinositol 4- kinase alpha among others.

RNA-Seq experiments became more frequently used in 2019 where shifts in transcriptional responses were measured in various *Leishmania* species and under more variable conditions. Patino et al. (2019) used RNA-Seq to analyze transcriptome profiles between antimony-resistant and antimony-sensitive *L. amazonensis* promastigotes. They identified a total of 723 differentially expressed genes between lines that were resistant or sensitive to trivalent sodium stibogluconate (Sb^*III*^). These genes encoded proteins associated with various biological processes, like adhesion, metabolism, cell cycle, autophagy, structural organization and stress response. Some mRNA encoding amastin proteins were overexpressed in resistant lines, whereas the downregulated protein-encoding genes encoded putative superoxide dismutase, other subset of the amastin family and transporter proteins, which included a folate/biopterin transporter, pteridine transporter and an ABC transporter (Patino et al., 2019).

Differential gene expression analysis between wild-type and Sb^*III*^-resistant *L. infantum* lines revealed 933 differentially expressed transcript including 837 upregulated and 96 downregulated transcripts (Andrade et al., 2020). Upregulated transcripts in the Sb^*III*^-resistant line were associated with protein phosphorylation, microtubule-based movement, ubiquitination, host–parasite interaction, cellular process, and other categories while downregulated transcripts were associated with ribonucleoprotein complex, ribosome biogenesis, rRNA processing, nucleosome assembly and translation (Andrade et al., 2020).

Global transcriptomics studies were similarly performed in *L. braziliensis* promastigotes and intracellular amastigotes. Espada et al. (2019) investigated the pathways related to intrinsic miltefosine tolerance in *L. braziliensis* clinical isolates and found upregulated Ros3 mRNA levels in sensitive strains compared to resistant ones and suggested that miltefosine transporter (MT)-Ros3 was responsible for the observed drug sensitivity in *L. braziliensis*. Drug efflux and compartmentalization were similar in resistant and sensitive strains and drug susceptibility did not correlate with SNPs in the MT-Ros3 (Espada et al., 2019).

Mechanisms underlying artemisinin resistance in *L. donovani* have been recently investigated using comparative genomic and transcriptomic analyses (Ghosh et al., 2020). A dependency on lipid and amino acid metabolism, a reduced DNA and protein synthesis leading to parasites in the quiescence state, and an active drug efflux have been observed in resistant isolates. Upregulated genes included those encoding cathepsin-L like protease, amastin-like surface protein, and amino acid transporter, while downregulated genes include ABCG2, pteridine receptor, adenylatecyclase-type receptor, phosphoaceylglucosamine mutase, and a collection of hypothetical proteins (Ghosh et al., 2020).

## Assessing the Effects of Environmental Variations

*Leishmania* parasites alternate between poikilothermic and homoeothermic hosts. Sudden temperature variations are natural events in their life cycles as the transmission from insect vector to the mammalian host includes a drastic increase over ambient temperature by more than 10 °C, which is in addition to the variation in temperature the insects experience in their habitats (Requena et al., 2012). The transcriptome of *L. major* promastigotes following a moderate heat shock from 26°C to 37°C was determined (Rastrojo et al., 2019). Following a moderate heat shock, Rastrojo et al. (2019) observed that the upregulated transcripts were heat shock proteins, amastigote-specific proteins and several hypothetical proteins. Downregulated transcripts, however, were associated with transporters, proteins involved in RNA metabolism or translational factors. Putative long non-coding RNAs were also identified among the differentially expressed transcripts and temperature-dependent changes in the selection of the SL addition sites were observed. Accordingly, alternative *trans-*splicing was suggested as an additional mechanism altering gene expression in *Leishmania* (Rastrojo et al., 2019).

Moreover, minor temperature shifts from 26°C to 24°C, 28°C, and 30°C) did not affect chromosomal ploidy but caused transcriptomic changes in *L. braziliensis* promastigotes grown *in vitro* (Ballesteros et al., 2020). Amastin surface-like proteins were downregulated under the three temperatures compared with the control (Ballesteros et al., 2020).

## Future Directions

Global RNA-Seq and transcriptome analysis has gained significant attention as a tool to unveil *Leishmania* species molecular biology and their pathogenicity, differentiation into various life stages and interplay with the host immune response (Dillon et al., 2015b; Shadab et al., 2019). The simultaneous analysis of the gene expression profiles of parasites and hosts is a critical step in our understanding of the disease (Dillon et al., 2015b; Masoudzadeh et al., 2020b). However, with the colossal amount of data generated, there is a need to “translate” these complex datasets into forms that can be exploited for drug and vaccine development.

Various species such as *L. tropica* remain understudied using RNA-seq, which mostly include data derived from *in vitro* cultures. Also, in order to perform an accurate comparative analysis using global RNA-Seq data, experimental design should be reproducible and cover various *Leishmania* species grown *in vitro* under similar well-defined conditions and infected in human macrophages similarly maintained under constant growth conditions and followed up in time course experiments to gain insights into the dynamic process of the infection. The outcome of such analysis should be further enriched and validated *in vivo* with transcriptomics data derived from promastigotes isolated directly from the sandfly gut and from amastigotes collected from induced lesions in mice models.

Reports suggesting only a moderate correlation between transcript abundance and cellular protein levels in *Leishmania* species requires further attention (de Pablos et al., 2019). Proteomics allow the analysis of expression levels, post-translational modifications, interactions, structure and subcellular distribution of proteins (Cox and Mann, 2007). Proteomics have been widely used to generate a proteome map providing an overall picture of gene expression at a given point in time (Sundar and Singh, 2016). In *Leishmania*, the comparison of proteomes has been employed to provide a better understanding of the parasite’s life cycle, host–pathogen interactions, protein–protein interactions and drug resistance mechanisms for different *Leishmania* species (Capelli-Peixoto et al., 2019). Several key candidates for vaccine development have also been identified through proteomics investigations (Sundar and Singh, 2018; Sundar et al., 2019).

Despite the low observed correlation between RNA and protein abundance in *Leishmania* (Leifso et al., 2007; de Pablos et al., 2019), proteomic technologies could complement genomics and transcriptome profiling studies to characterize specific gene products (Sundar and Singh, 2016). Furthermore, complementing genome sequence with transcriptomics and proteomics data enable more accurate assembly and annotation of newly sequenced genomes (Cox and Mann, 2007; Prasad et al., 2017).

Moreover, the control of mRNA stability, decay and translation in the near absence of transcriptional regulation is not well understood (de Pablos et al., 2016). Also, the dynamics and regulation of messenger ribonucleoprotein complexes (mRNPs) in *Leishmania* species or differences in translational regulation from ribosomal profiling of the mRNAs from each life cycle stage are not yet elucidated (de Pablos et al., 2016).

Potentially sinefungin could be used to examine gene transcription. Sinefungin is a naturally occurring nucleoside isolated from *Streptomyces griseolus* and *S. incarnates* bacteria with structural similarities with S-adenosyl-1-methionine (Bachrach et al., 1980; Dube et al., 1983; McNally and Agabian, 1992). Sinefungin has antitrypanosomal activity and inhibits SL *trans*-splicing in these organisms (Bachrach et al., 1980; Dube et al., 1983; McNally and Agabian, 1992; Pandarakalam et al., 2019).

The role of adenylate cyclase in *Leishmania* parasites residing inside insect hosts requires further investigations (Iantorno et al., 2017). Also, more studies should focus on the role of amastins in infection, survival and host-parasite interactions.

Another interesting target is to investigate the roles of non-coding RNAs (ncRNAs) in regulating *Leishmania* mRNA transcription (Castro et al., 2017; Ruy et al., 2019). ncRNAs emerge as key players in a variety of regulatory processes including mRNA processing, mRNA stability in addition to DNA replication, chromosome maintenance and transcriptional regulation in various organisms (Mattick, 2005; Morris and Mattick, 2014). In *L. major* and *L. donovani*, 26 and 30 putative ncRNAs were identified, respectively, with the majority arising from UTRs (Castro et al., 2017). The biological function of these ncRNA and their regulatory roles in transcription deserve further investigation.

Moreover, a detailed metatranscriptomics analysis *in situ* in the skin and in the insect vector covering the insect gut microbiota and the human skin microbiota, respectively, could aid in enriching our understanding of the factors that govern the complex host–parasite-microbiota interplay and their potential impact and relationship with the different forms of leishmaniasis, CL, VL, and MCL. Skin microbiota dysbiosis associated with CL requires further attention modulating the pathobiology of *Leishmania* infections and appears, importantly, as a potential novel therapeutic target (Gimblet et al., 2017). Notably, the mammalian skin and insect gut microbiota are also potentially relevant to trigger and modulate the shift from CL to VL (Gimblet et al., 2017). Moreover, the impact of LRV and other viruses, such as the Lymphocytic choriomeningitis virus (LCMV), on the pathobiology of various *Leishmania* species would also be of great interest to investigate with more integrative approaches and model systems (Ives et al., 2011; Crosby et al., 2015). Lymphocytic choriomeningitis virus, for instance, is a rodent-borne, Old World arenavirus that causes asymptomatic or mild, self-limited illness in otherwise healthy humans (Zhou et al., 2012). It is a known cause of aseptic meningitis and rarely fatal (Fischer et al., 2006). It has been reported that an increased recruitment of neutrophils and more severe lesions were observed in mice co-infected with *L. major* and LCMV (Crosby et al., 2015). More recently, a ssRNA *Leishmania*-infecting leishbunyavirus (*Lmar*LBV1) has been isolated from *Leishmania martiniquensis* (Grybchuk et al., 2020). Thus, the diversity and implications of viruses on leishmaniasis outcome and disease progression are other lines of research of great interest to investigate.

Gut microbes from the sandfly are egested into the host skin alongside *Leishmania* parasites at the bite sites (Dey et al., 2018). These have been shown to augment the severity of VL caused by *L. donovani* via inflammasome-derived IL-1β production (Dey et al., 2018).

Although much remains to be elucidated on the effects of skin microbiota and sandfly gut microbes on the progression of leishmaniasis, understanding the complex interactions between *Leishmania*, skin microbiota and the sandfly gut microbes in disease outcome appears as a potential therapeutic target and an important parameter to untangle the complex immunological cross-talks occurring between the parasite and the host skin and/or sandfly gut microbiota.

## Author Contributions

TS: conceptualization, investigation, and writing—original draft and review and editing. TS, ST, and RH: data curation, investigation, and writing—original draft and review and editing. All authors listed have made a substantial, direct and intellectual contribution to the work, and approved it for publication.

## Conflict of Interest

The authors declare that the research was conducted in the absence of any commercial or financial relationships that could be construed as a potential conflict of interest.

## Publisher’s Note

All claims expressed in this article are solely those of the authors and do not necessarily represent those of their affiliated organizations, or those of the publisher, the editors and the reviewers. Any product that may be evaluated in this article, or claim that may be made by its manufacturer, is not guaranteed or endorsed by the publisher.
